# Global research trends of Middle East respiratory syndrome coronavirus: a bibliometric analysis

**DOI:** 10.1186/s12879-016-1600-5

**Published:** 2016-06-07

**Authors:** Sa’ed H. Zyoud

**Affiliations:** Division of Clinical and Community Pharmacy, Department of Pharmacy, Faculty of Medicine and Health Sciences, An-Najah National University, Nablus, 44839 Palestine; Poison Control and Drug Information Center (PCDIC), Faculty of Medicine and Health Sciences, An-Najah National University, Nablus, 44839 Palestine

**Keywords:** Bibliometric, *Scopus*, Coronavirus, Middle East respiratory syndrome coronavirus, MERS-CoV

## Abstract

**Background:**

Middle East respiratory syndrome coronavirus (MERS-CoV) is a virus that causes severe viral pneumonia in humans, known to have a high mortality rate and a similarity in clinical symptoms with severe acute respiratory syndrome coronavirus. It was first isolated in Kingdom of Saudi Arabia (KSA) in 2012 and after that, MERS-CoV exhibited outbreaks in several regions of the world. This study aimed to assess the characteristics of publications involving MERS-CoV at global level by using a bibliometric analysis.

**Methods:**

*Scopus* database was searched on March 4, 2016 for MERS-CoV publications published between 2012 and 2015. It was performed on the same day in order to avoid the possible bias came from update on the database because the metrics are changing over time. All publication types were considered; however publications as errata were excluded. Analysis parameters include year of publication, publication type, patterns of international collaboration, research institutions, journals, impact factor, *h*-index, language, and times cited.

**Results:**

A total of 883 MERS-CoV research publications were published across the world. The MERS-CoV-associated publications were originated from 92 countries/territories, indicating the international spread of MERS-CoV research. The USA was the largest contributor, with 319 articles published over 4 years, followed by KSA (113 articles). The total number of citations for these publications has already achieved 8,015, with an average of 9.01 citations per each publication. The *h*-index for MERS-CoV-associated publications was 48. The USA also have the highest *h*-index (32), followed by KSA (26) and UK (22). Netherland produced the greatest proportion of publications with international research collaboration (72.7 %) followed by the UK (71 %) and Germany (69.1 %) out of the total number of publications for each country.

**Conclusions:**

There is a rapid increase in research activities related to MERS-CoV from 2012 to 2015. This study demonstrates that the MERS-CoV related literature has grown to be more extensive and global over the past 4 years. The bulk of publications in the field of MERS-CoV research are published by high-income countries such as the USA. Furthermore, the USA, the UK and KSA may have higher quality of articles according to the value of *h-*index.

## Background

Middle East respiratory syndrome coronavirus (MERS-CoV) is a virus that causes severe viral pneumonia in humans, known as having a high mortality rate and having a similarity in clinical symptoms with severe acute respiratory syndrome coronavirus [1–3]. It was first happened in Jordan in April, 2012 [4], and then the virus was isolated from a patients in Kingdom of Saudi Arabia (KSA) [5–7]. After that, MERS-CoV exhibited outbreaks in several regions of the world [8–12]. Although MERS-CoV is thought to be a zoonosis, probably camels act as a direct source of human MERS-CoV infection [13, 14], most cases are now due to human-to-human transmission [9, 11, 12, 15–17]. The virus was firstly nominated Human Coronavirus Erasmus Medical Center/2012 (HCoV-EMC/2012) [5], but after global consensus it was renamed MERS-CoV [18].

In order to evaluate its current impact on global scientific research production, a bibliometric analysis was performed using available information indexed at most common journals-indexing databases, such as *Scopus*. Bibliometric analysis examines the progress of any topic and offers a comprehensive assessment of scientific research trends. In recent years, bibliometric analysis has been extensively performed to assess scientific activities in many fields, including infectious diseases such as Mayaro virus fever [19], Zika virus [20], Ebola virus disease [21, 22], yellow fever disease [23], dengue [24], Malaria [25], leishmaniasis [26, 27], influenza [28], and John Cunningham virus [29]. To the best of knowledge of the author, there has been no bibliometric study about MERS-CoV research in the English literature. This study aimed to assess the characteristics and quality of articles involving MERS-CoV at global level. The study approach is employed to assess MERS-CoV related research characteristics such as countries, journals, research institutions, and citation habits.

## Methods

Publications used in this analysis were extracted from the *Scopus* database developed by Elsevier in the Netherlands. Scopus is a relatively large database compared to PubMed and Web of Science [30] which contains information for publications published in more than 21,500 titles of which 20,000 are peer-reviewed journals from more than 5,000 international publishers [31]. Selected publications included “Middle East respiratory syndrome coronavirus” or “MERS-CoV” or “Human Coronavirus Erasmus Medical Center/2012” or “HCoV-EMC/2012” as a part of its title, abstract, or keyword from 2012 till the date of December 31, 2015 All of these terms are used in one query. Publications as errata or publications which their scope was not related to MERS-CoV, and publications from 2016 were excluded from the study. Research indicators for the assessment of MERS-CoV research output were determined according to the methods used previously in similar bibliometric studies [32–37]. Analysis parameters include date (year) of publication, publication type, patterns of international collaboration, research institutions, journals, impact factor (IF), *h*-index, language, and times cited. International collaborations were considered when the paper affiliation contained different countries.

To measure the impact and/or productivity of the research published in the field of MERS-CoV, *h*-index and IF were used as bibliometric indicators for this evaluation. The *h*-index was introduced in literature in 2005 by Hirsch for the assessment of individual academic attainment [38]. The *h*-index can more truthfully represent the author’s or country’s achievement; a higher *h-*index shows that the publication׳s larger influential power. The IF of the journals as reported in Journal Citation Reports® (JCR) 2014 was used [39]. Only the ten top-ranked journals were considered and they were shown in descending order from 1st to 10th using the standard competition ranking (SCR), with the highest absolute number of publications ranked 1, as obtained on the day of data collection (March 4, 2016). Data collection and analysis were performed on the same day in order to avoid the possible bias came from update on the database because the metrics are changing over time. Parameters with the same number of publications were given the same rank number.

### Statistical analysis

The analysis was performed using descriptive statistics in Microsoft Excel 2007, and SPSS statistical software (SPSS for Windows, version 15, SPSS Inc Chicago, IL, USA). The analysis involved the calculation of relative frequencies, percentages, sum, and average.

## Results

A total of 883 MERS-CoV-associated publications were retrieved in the study search. Nine publications were published in 2012, 155 in 2013, 318 in 2014, and 401 in 2015. Around sixty percent of total share was published as original articles, 13.3 % as reviews, 9.5 % as editorial materials, 7.5 % as letters, and the remaining being note, erratum, and conference Paper. Among these articles, 829 (93.9 %) were written in English, 18 (2.04 %) were in Korean, 12 (1.4 %) were in Chinese, 10 (1.1 %) were in German, and the remainder of articles were in a variety of other languages such as French, Czech, Dutch, Greek, Polish, and Hungarian.

The MERS-CoV-associated publications were originated from 92 countries/territories, indicating the international spread of MERS-CoV research. Table 1 shows the 10 countries with the highest number of publications included in the *h*-indices. Out of 883 publications, the USA was the largest contributor, with 319 (36.1 %) articles published over 4 years; this was followed by KSA (113, 12.8 %), China (103, 11.7 %), and the UK (93, 10.5 %). The total number of citations for these publications has already achieved 8,015, with an average of 9.01 citations per each publication. The *h*-index for MERS-CoV-associated publications was 48. The USA also have the highest *h*-index (32), followed by KSA (26) and the UK (22). Netherland produced the greatest proportion of publications with international research collaboration (72.7 %) followed by the UK (71 %) and Germany (69.1 %) out of the total number of publications for each country (Table 1).

**Table 1:** Top ten countries/territories with scientific production on MERS-CoV research at *Scopus* (up to December 31, 2015)

SCR	Countries/territories	Total number of publications for the whole period (%)	h-index	Number (%) of publications with international collaboration
1st	United States	319 (36.1)	32	168 (52.7)
2nd	Saudi Arabia	113 (12.8)	26	76 (67.3)
3rd	China	103 (11.7)	19	63 (61.2)
4th	United Kingdom	93 (10.5)	22	66 (71.0)
5th	Hong Kong	71 (8.0)	18	47 (66.2)
6th	Germany	68 (7.7)	21	47 (69.1)
7th	Netherlands	55 (6.2)	21	40 (72.7)
8th	South Korea	39 (4.4)	4	4 (10.3)
9th	France	37 (4.2)	10	17 (45.9)
10th	Australia	27 (3.1)	6	13 (48.1)

*SCR* standard competition ranking

The MERS-CoV-associated publications were published in 384 different journals, but most frequently in these journals: *Journal of Virology* (46), *Emerging Infectious Diseases* (43), *Eurosurveillance* (36), and *The Lancet Infectious Diseases* (35); (Table 2). The IF for journals including top 10 cited MERS-CoV publications ranged from 1.859 to 45.217.

**Table 2:** The 10 most published journals

SCR^a^	Journal	Frequency (%)	IF ^b^
1st	*Journal of Virology*	46 (5.2)	4.439
2nd	*Emerging Infectious Diseases*	43 (4.9)	6.751
3rd	*Eurosurveillance*	36 (4.1)	5.722
4th	*The Lancet Infectious Diseases*	35 (4.0)	22.433
5th	*MBio*	34 (3.9)	6.786
6th	*The lancet*	24 (2.7)	45.217
7th	*International Journal of Infectious Diseases*	20 (2.3)	1.859
8th	*Virus Research*	17 (1.9)	2.324
9th	*Journal of the Korean Medical Association*	15 (1.7)	NA
9th	*Journal of Infectious Diseases*	15 (1.7)	5.997

*SCR* standard competition ranking, *NA* not available, *IF* impact factor

^a^ Equal journals have the same ranking number, and then a gap is left in the ranking numbers

^b^ The impact factor was reported according to the journal citation reports (JCR) 2014

Table 3 shows the top-cited publications published in the field of MERS-CoV [2, 5, 6, 18, 40–46]. The 10 most frequently cited articles have been cited more than 137 times from their initial publication year until March 4, 2016. The most frequently cited article (645 citations) was published by Zaki et al. in 2012 in the *New England Journal of Medicine* [5], followed by Assiri et al. [40] which was published in the *New England Journal of Medicine* in 2013. Majority of the top cited papers were published in journals with high IF (IF > 10).

**Table 3:** The top 10 cited publications

SCR^a^	Authors	Title	Year of publication	Source title	Cited by	IF^b^
1st	Zaki et al [5]	Isolation of a novel coronavirus from a man with pneumonia in Saudi Arabia	2012	*New England Journal of Medicine*	645	55.873
2nd	Assiri et al [40]	Hospital outbreak of middle east respiratory syndrome coronavirus	2013	*New England Journal of Medicine*	256	55.873
3rd	van Boheemen et al [45]	Genomic characterization of a newly discovered coronavirus associated with acute respiratory distress syndrome in humans	2012	*MBio*	217	6.786
4th	Raj et al [46]	Dipeptidyl peptidase 4 is a functional receptor for the emerging human coronavirus-EMC	2012	*Nature*	216	41.456
5th	Reusken et al [41]	Middle East respiratory syndrome coronavirus neutralising serum antibodies in dromedary camels: A comparative serological study	2013	*The Lancet Infectious Diseases*	190	22.433
6th	de Groot et al [18]	Middle east respiratory syndrome coronavirus (MERS-CoV): Announcement of the coronavirus study group	2013	*Journal of Virology*	172	4.439
7th	Assiri et al [2]	Epidemiological, demographic, and clinical characteristics of 47 cases of Middle East respiratory syndrome coronavirus disease from Saudi Arabia: A descriptive study	2013	*The Lancet Infectious Diseases*	150	22.433
8th	Bermingham et al [6]	Severe respiratory illness caused by a novel coronavirus, in a patient transferred to the United Kingdom from the Middle East, September 2012	2012	*Eurosurveillance*	148	5.722
9th	Guery et al [42]	Clinical features and viral diagnosis of two cases of infection with Middle East Respiratory Syndrome coronavirus: A report of nosocomial transmission	2013	*The Lancet*	139	45.217
10th	Memish et al [43]	Middle East respiratory syndrome coronavirus in Bats, Saudi Arabia	2013	*Emerging Infectious Diseases*	137	6.751
10th	Memish et al [44]	Family cluster of middle east respiratory syndrome coronavirus infections	2013	*New England Journal of Medicine*	137	55.873

SCR, Standard competition ranking; NA, Not available; IF, Impact factor

^a^ Equal articles have the same ranking number, and then a gap is left in the ranking numbers

^b^ The impact factor was reported according to the journal citation reports (JCR) 2014

The contribution of the 10 most productive institutes in MERS-CoV research from 2012 to 2015 is shown in Table 4. Among the top 10 institutes, 4 institutes are from the USA, 2 from KSA; and one from Hong Kong, Netherland, UK, China and Germany respectively. The *University of Hong Kong* had the maximum contribution in terms of the total volume of publications with 68 articles, followed by the Ministry of Health Saudi Arabia in KSA (63 articles) and *Erasmus University Medical Center* in Netherland (47 articles).

**Table 4:** The top 10 highly productive and influential institutions in research on MERS-CoV field

SCR^a^	Institution, country	No. of publications (%)
1st	The University of Hong Kong, Hong Kong	68 (7.7)
2nd	Ministry of Health Saudi Arabia, KSA	63 (7.1)
3rd	Erasmus University Medical Center, Netherland	47 (5.3)
4th	Universitat Bonn, Germany	37 (4.2)
5th	University College London, UK	36 (4.1)
6th	Centers for Disease Control and Prevention, USA	30 (3.4)
7th	The University of North Carolina at Chapel Hill, USA	28 (3.2)
8th	Fudan University Shanghai Medical College, China	27 (3.1)
8th	Alfaisal University, KSA	27 (3.1)
10th	New York Blood Center, USA	26 (2.9)
10th	Indiana University School of Medicine Indianapolis, USA	26 (2.9)

*SCR* standard competition ranking, *KSA* Kingdom of Saudi Arabia

^a^ Equal institutions have the same ranking number, and then a gap is left in the ranking numbers

## Discussion

The current study showed a rapid increase in research activities related to MERS-CoV in the past 4 years. The USA and KSA were the most productive countries. These results are not surprising because the USA has played an important role in fostering international cooperation on MERS-CoV research and control due to the potential risk that this represents globally. Another possible explanation for this is that the USA is the most prolific country for scientific research in general in previous bibliometric studies [19, 32–36]. It seems possible that these results are due to its size and economic strength. The ten most prolific countries that were involved in MERS-CoV research contains new nations different from the familiar global ranking [47]. Through the current study, KSA leadership in MERS-CoV global research (12.8 % of the total) clearly stands up, most likely due to the fact that it was in this country where the virus originally isolated [5] and several outbreaks have been reported in this country [10, 15, 48–51]. Other countries as those located in the Asia-Pacific such as China, and South Korea have also increased their scientific MERS-CoV research output in the last year owing their new outbreaks [8, 52–56].

In the current study, the most commonly language used in MERS-CoV research is English. Additionally, it is because English is now used extensively and is considered one of the most widespread language in the world [57], and the majority of journals indexed in *Scopus* are published in English.

The findings of this study recognize the publications associated with the most important developments in the field of MERS-CoV. This bibliometric analysis reveals that the most cited article in MERS-CoV is the 2012 paper by by Zaki et al [5], in *New England Journal of Medicine.* This article reported the first isolated MERS-CoV in June 2012 from a Saudi male aged 60 years [5]. The second most cited article was written in 2013 by Assiri et al [40]. The authors reported a total of 23 cases related to MERS-CoV infection in the eastern region of KSA. Furthermore, the 2 most highly cited articles were published in a relatively high-IF journal (*New England Journal of Medicine*), which has an IF of 55.873. Some previous study showed that the IF of the journal was the strongest marker for citations [58–60]. Findings of this study verify the close relationship between IF and citations, and that the most cited articles are usually published in journals that top the IF list, which also helps keep the high IF of these journals [60]. However, the *h-*index was launched in 2005 [38] which have been used to quantify of quality and impact of the scientific research output of a researcher, countries, institutions, and journals. The *h-*index was used in the current study for the top 10 countries. Of the top 10 countries, 5 had an *h-*index of 20 or more.

The large impact on scientific research output in reference to MERS-CoV research replicates its global influence as a potentially harmful disease. The most interesting finding of this study was that the total number of publications with the international collaboration for each country are a bit greater than that found in earlier bibliometric reports in different fields [33, 34, 36]. While these earlier bibliometric studies have described the importance of global collaboration, which considered as the most effective strategy to increase citation rates [36, 61–63]. Moreover, the global map of scientific collaboration networks and production lets researchers to contribute for implementation of new strategy for controlling MERS-CoV outbreaks to reduce morbidity and mortality related with such outbreaks [10, 40, 50]. Furthermore, collaborative research clearly demonstrates the prioritization in searches for a appropriate vaccine as well as effective medications for treatment of MERS-CoV [64, 65].

The most important limitation lies in the fact that the *Scopus* database was used to search for MERS-CoV. So publications indexed in none *Scopus*-cited journals were not studied. Another possible limitation to this study is the use of absolute number of citations in ranking the articles, instead of rankings based on average number of citations per year. Furthermore, the number of research output in 2015 may be rising because Scopus is still open for new journals issues from this year.

## Conclusions

Based on the *Scopus* database, the characteristics of the MERS-CoV research output from 2012 to 2015 are investigated by means of bibliometric methods. There is a rapid increase in research activities related to MERS-CoV from 2012 to 2015. This study demonstrates that the MERS-CoV related literature has grown to be more extensive and global over the past 4 years. The bulk of publications in the field of MERS-CoV research are published by high-income countries such as the USA. Furthermore, the USA, the UK and KSA may have higher quality of articles according to the value of *h-*index. These findings show the value of bibliometric method to illustrate global research trends of MERS-CoV. Thus, this study provides a helpful reference for medical virologists and epidemiologists, policy decision makers, academics, and MERS-CoV researchers. As MERS-CoV can be considered a recent emerged disease, and a new research topic, the study results characterize a ‘snapshot’ of this field at an early stage in its development.

## Abbreviations

HCoV-EMC/2012, Human Coronavirus Erasmus Medical Center/2012; IF, impact factor; IRB, institutional review board; KSA, Kingdom of Saudi Arabia; MERS-CoV, Middle East respiratory syndrome coronavirus; SCR, standard competition ranking.
